# Specific mindfulness traits protect against negative effects of trait anxiety on medical student wellbeing during high-pressure periods

**DOI:** 10.1007/s10459-021-10039-w

**Published:** 2021-03-06

**Authors:** E. Fino, M. Martoni, P. M. Russo

**Affiliations:** Department of Experimental, Diagnostic and Specialty Medicine (DIMES), Alma Mater Studiorum - University of Bologna, Via Giuseppe Massarenti 9, 40138 Bologna, Italy

**Keywords:** Medical student wellbeing, Trait anxiety, Mindfulness traits, Perceived stress, Psychosomatic problems, Sleep‐wake quality

## Abstract

Medical education is highly demanding and evidence shows that medical students are three times more susceptible to deteriorating physical and mental health than the average college student. While trait anxiety may further increase such risk, little is known about the role of trait mindfulness in mitigating these effects. Here we examine the protective role of specific mindfulness facets as mediators in pathways from trait anxiety to perceived stress, psychosomatic burden and sleep-wake quality in medical students, across repeated measurements throughout the first trimester of the school year. Preclinical medical students enrolled in the second year of the Medical School of University of Bologna completed self-report questionnaires examining personality traits as well as physical and psychological wellbeing. Data were collected at the beginning (Time 1: N = 349) and the end of the first trimester (Time 2: N = 305). As students approached the end of the trimester and upcoming exams, reported levels of perceived stress, psychosomatic problems and difficulties in wakefulness increased significantly compared to the beginning of the trimester. Mediation results showed that trait anxiety predicted such outcomes whereas the protective role of mindfulness facets in mitigating these effects was significant only at Time 2. Specific facets of Nonjudging of inner experience and Acting with awareness proved to be the most effective mediators. Findings highlight that the beneficial role of mindfulness facets in mitigating negative consequences of trait anxiety on medical student wellbeing is revealed in high-pressure periods and when self-regulation is needed the most. Cultivating awareness and nonjudgmental acceptance of one’s inner experiences is a crucial self-regulation resource that can help medical students sustain their wellbeing as they learn and throughout their high-pressure education and professional careers.

## Introduction

Medical education is among the most protracted of professional healthcare programs. Long hours of training and clinical practice, lack of sleep, heavy workload and great expectations placed on student’s shoulders are but a shortlist of the inherent pressures of medical school, which seem to mirror the hardships of future professional life. While necessary for students to obtain the required amount of professional knowledge and skills such factors have also been linked with negative consequences for student wellbeing, performance outcomes, dropout rates, medical errors and quality of patient care (Dyrbye et al., 2006; Fahrenkopf et al., 2008; Shanafelt et al., 2010; West et al., 2016). Disquieting evidence by studies around the world shows that medical students are three times more susceptible to deteriorating mental and physical health than the average college student (Beiter et al., 2015; Cuttilan et al., 2016; Dyrbye & Shanafelt, 2011, 2016; Heinen et al., 2017; Mihailescu & Neiterman, 2019; Zeng et al., 2019). Paradoxical as it may seem, it has been pointed out that preparing the doctors that will care for the sick is taking an incredibly high toll on their very own mental and physical resources, which is also reflected in significant costs for national healthcare and education systems (Kemp et al., 2019; Ripp et al., 2017). In recognition of the problem’s magnitude, a wide consensus is rising worldwide calling for actions to enhance medical student well-being as they learn, by supporting students to develop along the professional skills, the necessary self-care capacities that will sustain their wellbeing throughout their high-pressure medical education and professional careers (Kemp et al., 2019; Ripp et al., 2017).

In this perspective, it is crucial to identify individual traits that may contribute to accentuate or attenuate the risk of deteriorated wellbeing outcomes in medical students. Traits are understood as relatively stable characteristics that mark significant differences between individuals. For instance, individuals that are high in trait anxiety have a pronounced tendency to attend to, experience and report negative emotions such as fears, worries and anxiety across many situations (Gidron, 2013). This pattern of behavior has been associated with the personality dimension of neuroticism (Costa & McCrae, 1992) and both trait anxiety and neuroticism stand out as individual differences that play an important role in physical and mental health. Individuals that are high in neuroticism tend to experience and react more negatively to stressful situations and have a heightened stress response, which leads to hyperarousal of the autonomic nervous system and hypothalamic-pituitary-adrenal axis overactivation, thus developing a predisposition to stress related conditions such as cardiovascular disease, psychosomatic disorders and sleep disturbances (Azad et al., 2015; Puig-Perez et al., 2016). Heightened anxiety and neuroticism traits are often associated with poor sleep quality, which in turn may further increase the vulnerability to psychological distress. Sleep is a critical aspect of our health and well-being and maintaining good sleep-wake quality is essential for optimal cognitive functioning, emotion regulation and physiological processes (Krause et al., 2017; Raven et al., 2017; Walker, 2009).

While this body of research has concentrated on the negative consequences of individual traits on physical and psychological wellbeing, other traits have been reported to play a more positive role in this regard. For instance,  trait mindfulness has been found to protect against maladaptive behaviors among university students with benefits for psychological health outcomes (Kabat-Zinn, 2003; Keng et al., 2011; McConville et al., 2017; Single et al., 2019; Tomlinson et al., 2018). As a trait, mindfulness has been conceptualized as a multifaceted disposition that at its core is characterized by nonjudgmental awareness of the present moment experience. From the theoretical prospective, trait mindfulness can be conceptualised as a marked disposition, at the attentional level, to be aware of the present moment (Bostanov et al., 2012) and at the meta-cognitive level, to be nonjudgmental and accepting of inner experience (Bishop et al., 2004). Several instruments have been specifically designed to capture the multiple components of this construct, the most largely used among them being the Five Facet Mindfulness Questionnaire (FFMQ; Baer, Smith, Hopkins, Krietemeyer & Toney, 2006). FFMQ operationalizes trait mindfulness as a set of five facets: (1) Observing internal and external experiences such as sensations, cognitions, emotions, sounds, and smells; (2) Describing internal experiences with words; (3) Acting with awareness instead of behaving mechanically while attention is focused elsewhere; (4) Non-judging and taking a non-evaluative stance toward the inner experience; (5) Non-reacting to inner experience and allowing thoughts and feelings to come and go (Baer et al., 2006). According to such conceptualization, present-moment attention and awareness allows for the observation of one’s own sensations, thoughts and emotions in a way that decouples their experience from the self and this very process of cognitive decentering is believed to enhance one’s ability to understand what one is experiencing (Shapiro et al., 2006) and to facilitate the nonjudgmental acceptance of (especially unpleasant) internal experiences, which enhances emotion regulation while reducing maladaptive responses (Hofmann et al., 2010). Therefore, individuals high in mindfulness traits have the ability to slow down automatic reactions in stressful situations, thus mitigating emotional reactivity and buffering against negative effects of stressors on psychological wellbeing.

While research has amply examined levels of mindfulness as negatively related to neuroticism, stress, insomnia, anxiety, and depression (e.g., Bhambhani & Cabral 2015; Chi et al., 2018; Hulsheger et al., 2015; Hofmann et al., 2010; Soysa & Wilcomb, 2015), there is little research that explores the impact of specific components of mindfulness on psychophysical wellbeing. A few studies adopting the multifaceted construct of trait mindfulness have highlighted the difference between facets in predicting psychological wellbeing (Calvete et al., 2017; Medvedev et al. 2018; Reese et al., 2015; Single et al., 2019). In particular, the facets operating at the meta-cognitive level of awareness (i.e., Nonjudging of inner experience, Acting with awareness and Nonreacting to inner experience) are strongly and negatively related with anxiety and depression (Alleva et al., 2014; Barnes & Lynn, 2010; Barnhofer et al., 2011; Cash & Whittingham, 2010; Desrosiers et al., 2013; Petrocchi & Ottaviani, 2016; Raphiphatthana et al., 2016), while, contrasting findings have been reported on facets such as Observing, most concerned with attention allocation, suggesting that sometimes an increased self-focus on internal processes might have maladaptive consequences on psychological well being. For instance, Observing is shown to be associated with higher levels of anxiety and psychological distress (Adams et al., 2012; Baer et al., 2008; Barnes & Lynn, 2010; Coffey et al., 2010; Desrosiers et al., 2013). With respect to Describing of inner experiences, research showing it is the most unrelated facet with any psychological wellbeing variable. Given that mindfulness facets do not operate in the same way, and that the mechanisms by which mindfulness might lead to beneficial outcomes still need to be fully clarified (Baer et al., 2008), an examination of complex constructs at the facet level is essential for clarifying their relationships with other variables (Sugiura & Sugiura, 2014).

The present study aimed to extend previous research on the five facets of mindfulness by examining their role in pathways from trait anxiety to perceived stress, psychosomatic problems and sleep-wake quality in medical students. We investigated this issue across repeated measurements throughout the first trimester of the school year and hypothesized that reported levels of perceived stress, psychosomatic symptoms and sleep-wake quality would be worsening from the beginning (Time 1) toward the end of the semester, nearing the exams period (Time 2). We also expected trait anxiety to influence perceived stress, psychosomatic burden and sleep-wake quality in medical students during the period prior to exams (Time 2). In addition, we hypothesised that only mindfulness facets that are mostly concerned with meta-cognitive level awareness like Nonjudging, Acting with awareness and Nonreacting would mediate these effects.

## Method

### Participants

**Fig. 1:**
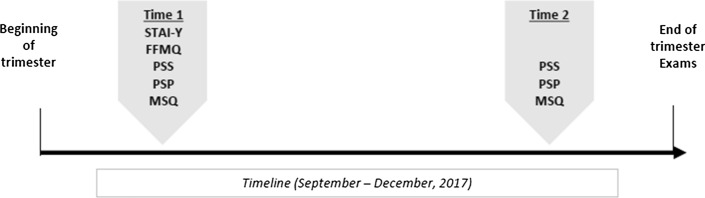
Timeline of student assessment along the trimester. *Note*: STAI-Y trait anxiety questionnaire, FFMQ the five facets mindfulness questionnaire, PSS the perceived stress scale, PSP the PsychoSomatic problems scale, MSQ the mini sleep questionnaire

Medical students (mean age 20.47 ± 1.90) attending the second year of the Medical School of University of Bologna participated in the study. Data were collected from self-reports distributed via email at two time points during the period from September 2017 to December 2017, at the beginning (Time 1: N = 349) and at the end of the trimester, prior to the exams (Time 2: N = 305). A timeline description of the data collection is provided in Fig. 1. Students were recruited through a psychology course and received course credit for their participation. Participants could respond to the questionnaires at their own pace and typically took about 20 min to complete all sections. Response rates ranged between 90 to 88% of all students attending the second year of Medical School, with a 12% of dropout between Time 1 and Time 2 points of data collection. Ethical approval of the study was granted by the Institutional Review Board of University of Bologna and all participants provided written informed consent.

### Measures

*Trait anxiety* was measured at the beginning of the semester (Time 1) through the State Trait Anxiety Inventory (STAI-Y; Spielberger et al., 1970). The STAI-Y is a unifactorial scale consisting of 20 items for assessing trait anxiety including items such as “I worry too much over something that really doesn’t matter” and “I am content; I am a steady person.” All items are rated on a 4-point scale (e.g., from “almost never” to “almost always”) with higher scores indicating greater anxiety. The Cronbach’s alpha in the present study was 0.87.

To measure *trait* *mindfulness* we used the Five Facets Mindfulness Questionnaire (FFMQ; Baer et al., 2006) which was administered at Time 1. The FFMQ consists in 39-items which load into five factors otherwise called mindfulness facets: “Observe” (e.g., “I notice visual elements in art or nature, such as colors, shapes, textures, or patterns of light and shadow”), “Describe” (e.g., “It’s hard for me to find the words to describe what I’m thinking”, reversed), “Acting with awareness” (e.g., “It seems I am “running on automatic” without much awareness of what I am doing”, reversed), “Nonjudging of experience” (e.g., “I believe some of my thoughts are abnormal or bad and I shouldn’t think that way”, reversed), and “Nonreacting to inner experience” (e.g., “I perceive my feelings and emotions without having to react to them”). Participants rated themselves on a 5-point Likert scale ranging from 1 = never to 5 = always. All facets contain eight items each except Nonreacting (seven items) and single scores for each of the facets were calculated. Cronbach’s alphas obtained in the present study were 0.76 for Observing; 0.90 for Describing; 0.88 for Acting with awareness; 0.89 for Nonjudging of inner experience and 0.77 for Nonreacting.

The Perceived Stress Scale (PSS; Cohen et al., 1983) was used to measure *perceived stress.* The scale consists of 10 items evaluating an individual’s appraisal of their life as stressful (i.e. unpredictable, uncontrollable and overloading) during the past week. Item examples include, ‘‘How often have you felt nervous or stressed?’’ and ‘‘How often have you felt confident about your ability to handle your personal problems?’’ and load into one factor. Measurements were taken at the beginning (Time 1, Cronbach’s alpha = 0.90) and end of the trimester (Time 2, Cronbach’s alpha = 0.89), respectively in order to evaluate the fluctuations of perceived stress along the trimester. Participants rated their experience on a five-point Likert scale from 0 = never to 4 = very often. Scores range from 0 to 40 with higher scores indicating greater overall distress.

*Psychosomatic problems* were measured through the PsychoSomatic Problems Scale (PSP; Hagquist, 2008) which is a widely used and validated self-report measure of psychosomatic problems in adolescents and young adults. Subjects are asked to indicate the extent to which they have experienced a series of somatic problems during the school year, for instance “suffered from stomach aches”. Responses are scored on a 5-point scale from 0 = never to 4 = always with score ranging from 0 to 24, where higher scores indicate a higher burden of psychosocial problems. The scale is unifactorial and was administered at both time-point measurements, Cronbach’s alpha coefficients at Time 1 and Time 2 were 0.77 and 0.75 respectively.

*The subjective quality of sleep and wake* was measured with the Italian version of the Mini Sleep Questionnaire (MSQ; Fabbri et al., 2006; Natale et al., 2014) which is a widely used tool for screening sleep disorders. The MSQ is a ten-item self-report scale measuring difficulty of sleep and wake and consists of two sub-scales investigating both sleep quality and daytime sleepiness. The sleep quality dimension includes items investigating features such as difficulties in falling asleep, presence of nighttime awakenings, etc., with scores ranging from 5 to 35. The wake quality dimension covers areas such as daytime sleepiness, presence of headache at morning awakening, with scores ranging from 4 to 28. Responses are scored on a 7-point scale from 1 = never to 7 = always, with higher scores on the two dimensions corresponding to a lower quality of sleep and wake. Values ≥ 16 for the dimension of sleep and ≥ 14 for the dimension of wake indicate marked difficulties of sleep and wake. Based on the Italian validation study (Natalet al., 2014) the item on snoring did not load on any of the two factors, hence the calculation of the sleep and wake quality scores in the present study was performed based on 5 and 4 items respectively. Cronbach’s alpha for measurements at Time 1 and Time 2 were 0.79 and 0.80 for quality of sleep and 0.80 and 0.82 for quality of wakefulness.

### Statistical analysis

**Fig. 2:**
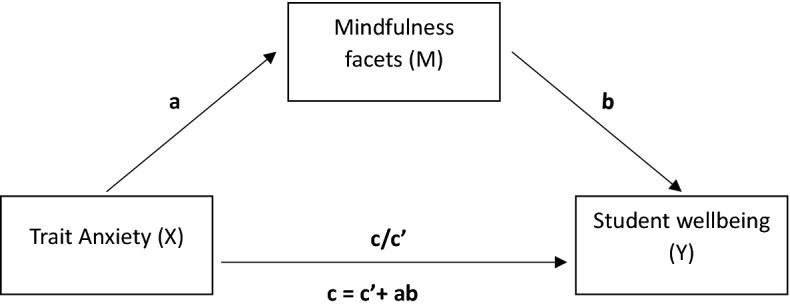
The mediation model: mindfulness facets (M) operate mediationally between trait anxiety (X) and student wellbeing (Y). Total effect is indicated by c. Direct effect is indicated by c′. Indirect effect is indicated by the path ab

Descriptive statistics were used to describe the sample. To identify potential covariates to include in subsequent analyses, differences between genders were analyzed by Bonferroni corrected t-tests and correlation coefficients were computed to evaluate associations between measures across time point measurements. To assess potential fluctuations over time, t-tests (and Wilcoxon Signed-rank test for non-normally distributed variables) were performed between measures evaluated at Time 1 and Time 2. Figure 2 represents the mediation model used in our main analysis with mindfulness facets set as mediator (M) in the relationship between trait anxiety (X) and student wellbeing variables (Y) of perceived stress, psychosomatic problems and sleep-wake quality. There is an open debate about whether and in which cases structural equations models (SEM) may be better suited for detecting indirect effects. While SEM increases the accuracy of measurement estimates it does so at the cost of reduced power and increased standard errors (Ledgerwood & Shrout, 2011). As low measurement error is essential when conducting mediation analyses (Aiken & West, 1991; Kenny & Judd, 2013) strong measurement reliability is important. Reliabilities of the scales in the present study are good to excellent. Considering our data characteristics, the bias-corrected and accelerated bootstrapping method by Preacher and Hayes (2008) was preferred. Separate multiple mediation model analysis using PROCESS macro (Hayes, 2013; Hayes & Preacher, 2014, model 4), were performed with 10,000 samples bootstrapping and bias corrected confidence intervals. In this procedure, a sample of cases from the complete data set is selected and the effects are determined in the resamples to generate the bootstrapping sampling distributions. It is a non-parametric test and bias-corrected for variables that are not normally distributed. Total effects, direct effects and indirect effects are estimated by means of ordinary least squares regression analyses, separately for each of the dependent variables. The effect of the independent variable (trait anxiety) is displayed in the total effect, and when controlling for the mediator variable (mindfulness facets) it is indicated in the direct effect. Most relevant for the mediation hypothesis, the indirect effect comprises the path over the mediator (mindfulness facets). This approach provides standardized betas for the indirect effect and a 95% bias corrected confidence interval for the size of the indirect path is generated. Following guidelines by Shrout and Bolger (2002), if the values between the upper and lower confidence limits do not include zero, this indicates a statistically significant mediation effect. By providing accelerated confidence intervals the bootstrapping method mitigates power problems and constitutes more accurate type I error rates, thus offering a more reliable estimation than the traditional Sobel test (Sobel, 1986) or the causal step method by Baron and Kenny (1986) for testing indirect effects. All statistics were performed using IBM SPSS Statistics for Windows (IBM Corporation, 2012) and the macro PROCESS (Hayes, 2013) with significance level set at *p* < .05.

## Results

### Preliminary analyses

Data from two students reporting that had previously received some kind of mindfulness-based training were excluded from final analysis. Gender differences examined in light of their role in anxiety and wellbeing variables were found for trait anxiety, perceived stress, psychosomatic problems, quality of wake and mindfulness facets of Nonjudging and Nonreacting. As the data was skewed (not normally distributed) the most appropriate statistical test was Wilcoxon Signed-rank test. Across time-point measurements, female students showed higher levels of anxiety, perceived stress, psychosomatic problems and poorer quality of wake compared to males. Females also showed lower levels in mindfulness facets of Nonjudging and Nonreacting than male students. Table 1 reports mean scores and standard deviations of all variables measured at both time points. As expected, there was a significant increase from Time 1 to Time 2 in reported levels of perceived stress (median_Time1_ = 19; median_Time2_ = 22; Z = − 6714, *p* < .0001), psychosomatic problems  (median_Time1_ = 19; median_Time2_ = 22; Z = − 3511, *p* < .0001) and quality of wakefulness (median_Time1_ = 13; median_Time2_ = 14; Z = − 4159, *p* < .0001). On the contrary, no significant difference was reported on quality of sleep between two time point measurements (Z =  − 0.945, *p* = .345). Notably, levels of perceived stress and psychosomatic problems were moderate to high at both time points and almost half of the sample reported poor quality of wakefulness according to recommended cutoff points (see Table 1).

**Table 1:** Descriptive characterization (Mean, SD, N, %) of the entire sample and of male and female students separately, with variables measured at the beginning (Time 1) and end of the trimester (Time 2)

	Total sample (n = 349)	Male students (n = 173)	Female students (n = 176)	*p*
*Beginning of the trimester—Time 1*				
Trait anxiety (STAI-Y)	44.99 (10.4)	42.86 (10.8)	47.09 (9.6)	**< 0.001**
Observing (FFMQ)	26.32 (5.7)	25.99 (5.7)	26.76 (5.7)	0.247
Describing (FFMQ)	27.20 (6.4)	27.25 (6.3)	27.37 (6.6)	0.873
Acting with awareness (FFMQ)	27.82 (5.9)	27.74 (5.8)	27.91 (5.9)	0.801
Nonjudging (FFMQ)	24.31 (7.1)	25.30 (6.8)	23.24 (7.4)	**0.014**
Nonreacting (FFMQ)	20.11 (4.4)	21.01 (3.9)	19.49 (4.7)	**0.003**
Perceived stress (PSS)	19.45 (7.0)	18.02 (6.9)	20.87 (6.9)	**< 0.001**
PsychoSomatic problems (PSP)	19.03 (5.1)	17.78 (5.1)	20.18 (4.7)	**< 0.001**
Sleep quality (MSQ)	12.37(5.8)	12.41 (5.7)	12.33 (5.8)	0.891
Score ≤ 15	254 (72.1)	124 (72.1)	130 (73.4)	
Score ≥ 16	95 (27.9)	48 (27.0)	46 (26.6)	
Wakefulness quality (MSQ)	13.04 (4.7)	12.16 (4.2)	13.90 (5.0)	**0.001**
Score ≤ 13	199 (57)	112 (56.3)	87 (43.7)	
Score ≥ 14	150 (43)	61 (40.7)	89 (59.3)	

	Total sample (n = 305)	Male students (n = 143)	Female students (n = 162)	*p*
*End of the trimester—Time 2*				
Perceived stress (PSS)	22.05 (7.0)	20.64 (6.9)	23.18 (7.1)	**0.002**
PsychoSomatic problems (PSP)	19.81 (5.1)	18.42 (4.9)	20.90 (4.9)	**< 0.001**
Sleep quality (MSQ)	12.26 (5.9)	11.66 (5.5)	12.34 (5.9)	0.313
Score ≤ 15	211 (69.9)	107 (75.0)	108 (66.6)	
Score ≥ 16	94 (29.1)	36 (25.0)	54 (33.3)	
Wakefulness quality (MSQ)	14.07 (5.1)	12.82 (4.6)	15.01 (5.2)	**< 0.001**
Score ≤ 13	160 (52.4)	84 (52.0)	68 (42.0)	
Score ≥ 14	145 (47.6)	59 (48.0)	94 (58.0)	

*STAI-Y* trait anxiety questionnaire, *FFMQ* the five facets mindfulness questionnaire, *PSS* the perceived stress scale, *PSP* the PsychoSomatic problems scale, *MSQ* the mini sleep questionnaire

Significant differences are indicated in bold

Furthermore, all variables were significantly correlated in the predicted directions (see Table 2). Trait anxiety was strongly and positively related with perceived stress, psychosomatic problems and sleep-wake quality at both time-points. It is noteworthy that specific mindfulness facets were not equally related with trait anxiety and wellbeing variables: Nonjudging and Acting with awareness were the most strongly and negatively correlated with trait anxiety, perceived stress, psychosomatic symtoms and difficulties in sleep and wake, whereas Observing was positively related with trait anxiety, perceived stress, psychosomatic symtoms and sleep-wake problems, while Describing showed little or no correlation with dependent variables.

**Table 2:** Correlations between measures of trait anxiety (STAI-Y), trait mindfulness (FFMQ), perceived stress (PSS), psychosomatic symptoms(PSQ), sleep and wake quality (MSQ) with the last four variables measured at the beginning (Time 1) and end of the trimester (Time 2)

		1	2	3	4	5	6	7	8	9	10	11	12	13	14
1	Trait anxiety (STAI-Y)	1													
2	Observing (FFMQ)	.14	1												
3	Describing (FFMQ)	− .18*	.12	1											
4	Acting with awareness (FFMQ)	− .47*	− .14	.21*	1										
5	Nonjudging (FFMQ)	− .56*	− .20*	.05	.36*	1									
6	Nonreacting (FFMQ)	− .38*	.08	.15*	.04	.25*	1								
7	Perceived stress (Time 1)	.71*	.08	− .16*	− .39*	− .47*	− .34*	1							
8	Perceived stress (Time 2)	.65*	.13	− .17*	− .45*	− .58*	− .39*	.62*	1						
9	PsychoSomatic problems (Time 1)	.62*	.13	− .06	− .33*	− .39*	− .21*	.68*	.48*	1					
10	PsychoSomatic problems (Time 2)	.50*	.20*	-0.06	− .40*	− .46*	− .19*	.48*	.66*	.64*	1				
11	Sleep quality (Time 1)	.38*	.19*	0.02	− .22*	− .23*	− .15	.36*	.28*	.61*	.43*	1			
12	Wakefulness quality (Time 1)	.48*	.17*	-0.01	− .27*	− .34*	-0.07	.45*	.41*	.59*	.53*	.54*	1		
13	Sleep quality (Time 2)	.33*	.16*	-0.01	− .27*	− .29*	− .13	.27*	.32*	.48*	.60*	.64*	.40*	1	
14	Wakefulness quality (Time 2)	.44*	.12	-0.08	− .35*	− .40*	-0.1	.45*	.54*	.52*	.71*	.36*	.65*	.51*	1
	Mean	44.99	26.33	27.20	27.82	24.32	20.12	19.46	22.05	19.29	19.82	12.37	13.04	12.26	14.07
	SD	10.45	5.71	6.45	5.96	7.14	4.44	7.07	7.06	5.31	5.15	5.80	4.78	5.94	5.15

*STAI-Y* state trait anxiety questionnaire, *FFMQ* the five facets mindfulness questionnaire, *PSS* the perceived stress scale, *PSP* the PsychoSomatic problems scale, *MSQ* the mini sleep questionnaire

Significant correlations are highlighted in bold. *The correlation is significant at 0.01 level (2-tailed)

### Mediation analysis

#### Total, direct and indirect effects of trait anxiety on student wellbeing measures: the mediating role of mindfulness facets

Multiple mediation analysis were performed separately to test the role of mindfulness facets in mediating the relationship between trait anxiety with perceived stress, somatic symptoms and sleep-wake quality at the beginning (Time 1) and the end of the trimester (Time 2) respectively. Given the significant gender differences found consistently across time-point measurements, gender was included in the model as a covariate.

No mediation effect was observed for the models considering perceived stress, psychosomatic problems and sleep-wake quality measured at the beginning of the trimester (Time 1).

On the other hand, the total effects of the boot-strapped mediation analyses with dependent measures assessed at the end of the trimester (Time 2) indicated a strong relationship between trait anxiety with perceived stress (R^2^ = 0.55; MSE = 23.38, F_6,299_= 59.62, *p* < .0001); psychosomatic symptoms (R^2^ = 0.33, MSE = 17.93, F_6,299_= 24.05, *p* < .0001) and quality of wake (R^2^ = 0.25, MSE = 20.19, F_6,299_ = 15.70, *p* < .0001). A strong relationship was also evidenced between mindfulness facets of Nonjudging, Acting with awareness and Nonreacting with the dependent variables (see Table 3 for direct effects and associated 95% confidence intervals).

**Table 3:** Estimated coefficients for mediation model of mindfulness facets

Total sample (N = 231)	Total effect				Direct effect					Indirect effect Nonjudgment of inner experience												
	SE		95% CI		SE			95% CI		BootSE			95% CI									
Perceived stress	**0.653**	0.030	[0.389 0.508]		**0.329**		0.037	[0.154 0.298]		0.166		0.030	[0.107 0.224]									
PsychoSomatic problems	**0.498**	0.025	[0.196 0.294]		**0.268**		0.032	[0.068 0.195]		0.126		0.033	[0.061 0.192]									
Quality of wakefulness	**0.442**	0.026	[0.166 0.267]		**0.302**		0.034	[0.081 0.215]		0.106		0.039	[0.033 0.185]									
Quality of sleep	**0.334**	0.031	[0.126 0.246]		**0.210**		0.041	[0.035 0.198]		0.061		0.043	[− 0.024 0.148]									

Total sample (N = 231)	Indirect effect Acting with awareness				Indirect effect Nonreacting to inner experience																	
	BootSE			95% CI	BootSE			95% CI														
Perceived stress	0.092		0.029	[0.040 0.150]	0.066		0.019	[0.031 0.105]														
PsychoSomatic problems	0.095		0.048	[0.041 0.152]	0.009		0.021	[− 0.032 0.052]														
Quality of wakefulness	0.068		0.033	[0.006 0.134]	− 0.034		0.025	[− 0.085 0.015]														
Quality of sleep	0.057		0.036	[− 0.013 0.129]	0.007		0.025	[− 0.042 0.059]														

All confidence intervals generated with bias corrected and accelerated bootstrapping (N = 10,000). All findings in bold are significant at *p* < .0001.

*Total effect* the effect of trait anxiety

*Direct effect* the effect of trait anxiety controlling for mindfulness facets

*Indirect effect* the path via mindfulness facets

More importantly, for the mediation hypothesis, there was a decrease in the effect of trait anxiety in the model that included mindfulness facets indicating a mediation effect for dependent measures of perceived stress, psychosomatic problems and quality of wakefulness. The significance of the indirect effects (i.e. the pathway of trait anxiety on dependent measures via mindfulness facets) provided further evidence for Nonjudging and Acting with awareness, as successful mediators in this relationship (see Table 3 for estimated standardised coefficients). Observed ratios of indirect to total and direct effect indicate a medium effect of Nonjudging of inner experiences in mediating the relationship between trait anxiety with perceived stress, psychosomatic problems and quality of wakefulness, followed by a smaller effect size of Acting with awareness in mediating this relationship. Nonreacting had a small effect in negatively mediating the effect of trait anxiety on perceived stress but not on psychosomatic problems or quality of wakefulness.

## Discussion

This study examined the contribution of specific mindfulness facets in protecting against negative consequences of trait anxiety on student physical and psychological wellbeing in the context of medical education. The present data allow to gain a deeper understanding of the fluctuations in levels of perceived stress and psychosomatic problems in medical students, the impact of trait anxiety and the mediating role of specific facets of mindfulness in this relationship.

In line with previous research showing deteriorated health and wellbeing outcomes in medical students (Beiter et al., 2015; Cuttilan et al., 2016; Heinen et al., 2017; Mihailescu & Neiterman, 2019; Zeng et al., 2019) we found moderate to high levels of perceived stress and psychosomatic symptoms among the students across time-point measurements during the trimester, while poor quality of wakefulness was observed in almost half of the sample. As expected, there was an increase in levels of perceived stress, psychosomatic problems and difficulties in wakefulness towards the end compared to the beginning of the trimester, further corroborating literature on the pressures of medical education and potential impact on student wellbeing (Dyrbye & Shanafelt, 2011, 2016; Kemp et al., 2019). Such pressures are not borne equally by male and female students though, as indicated by the significant gender differences revealing females to be more prone to poor wellbeing outcomes compared to male students. Females also scored higher than males in trait anxiety and lower in mindfulness facets of Nonjudging and Nonreacting to experiences, suggesting a higher anxiety susceptibility in this subgroup, which is in line with independent studies on lay persons and nursing student samples (Dyrbye et al., 2006; Fino et al., 2018; Soysa & Wilcomb, 2015; Zeng et al., 2019).

Results of mediation analyses, accounting for the effect of gender were significant for perceived stress, psychosomatic symptoms and qualify of wakefulness. A full mediation model nevertheless was not obtained, as the direct effects of trait anxiety were still significant even after mindfulness facets were included in the model, indicating that multiple factors likely influence the association between trait anxiety and student wellbeing. Our main hypothesis regarding the mediating role of trait mindfulness in this relationship was confirmed in function of the time period and for specific mindfulness facets. The mediational role of mindfulness was significant nearing the exam period, where there was an increase in stress and psychosomatic problems as well as a deterioration in wakefulness quality among medical students. Such findings support the notion that, whereas mindfulness is an essential resource as it relates to self-regulatory capacity in general, its beneficial role in mitigating anxiety’s negative consequences on student psychophysical wellbeing may be specifically revealed when under pressure and when self-regulation is needed the most (Chambers et al., 2009). This further extends the literature on benefits of mindfulness for physical and psychological wellbeing (Hulsheger et al., 2015; Keng et al., 2011, Soysa & Wilcomb, 2015; Tomlinson et al., 2018).

With respect to sleep-related outcomes, the mediation effect of mindfulness facets in pathways from trait anxiety was significant only for quality of wakefulness but not for quality of sleep. Interestingly in our study, sleep quality was the only dependent variable that did not seem to worsen during the trimester. This means that the higher-pressure period right before the exams seems to have an impact more on the daytime functioning (i.e., daytime sleepiness) of our sample rather than on disturbed sleep patterns (i.e., difficulty of falling of asleep, night-time awakening). This could explain why the mediation effect of mindfulness facets did not result significant on this variable.

Another relevant contribution of the present study that extends current literature is that the mediation effects differed between specific facets of mindfulness. Our findings highlight the sizable contribution of Nonjudging of inner experiences followed by a smaller effect of Acting with awareness in mitigating effects of trait anxiety on perceived stress, psychosomatic problems and quality of wakefulness. By holding a non-judgmental stance towards one’s experience, one is less likely to engage in negative appraisals of the experience and thus one would be less prone to exhibit intense negative emotional reactions. Moreover, being non-critical and non-judgmental towards one’s thoughts and feelings may foster self-compassion and acceptance, which have been shown to be associated with positive affect. With respect to Acting with awareness, it may attenuate anxiety through reducing attentional resources utilized in negative appraisals and by directing attention to the present activity, one may also have less mental capacity to be hypervigilant, which has been shown to contribute to hyperarousal (Vanden Bogaerde et al., 2011). We found a small effect for Nonreacting to inner experiences in negatively mediating the relationship between trait anxiety on perceived stress, indicating that the ability to let one’s feelings come and go may be important in modulating stress related effects but not enough to also mediate those with regard to psychosomatic problems and quality of wakefulness. In line with our expectations and with previous findings (Baer et al., 2008; Coffey et al., 2010) present results showed no mediation effect for mindfulness facets of Observing and Describing, which supports the notion that while crucial to directing attentions and enhancing one’s awareness and capacity to describe once’s experiences at the present moment these two facets alone are not able to modulate trait anxiety.

While the pattern of results is clear and consistent with previous literature, certain limitations should be acknowledged. First, conclusions regarding causality may be limited by the single-site nature of the study, the exclusive reliance on self-report questionnaires and the fact that dependent measures were assessed only at two time-points. Secondly, in order to measure changes over time due to variations of the mediator, it is necessary that future studies include an experimental manipulation of the mediator (perhaps through mindfulness-based interventions) and gather data in longer time sequences in order to elucidate long-term consequences of trait anxiety on student wellbeing. Thirdly, mindfulness facets did not fully mediate the effect of trait anxiety on wellbeing variables, and therefore future studies should include other potentially mediating internal (i.e., differences in self-compassion) and external (i.e., medical education related) factors that are not assessed in the present study. For example, studies should consider changes in students’ levels of stress and psychosomatic problems and sleep-wake quality throughout and as a result of specific education programs and training modules that may be particularly challenging (e.g., clerkships, specific specialization programs, etc.).

Despite these limitations the present work contributes to shedding light into the relationship between trait anxiety and wellbeing outcomes in medical students. Importantly, the protective role of specific mindfulness facets was evidenced when it was needed the most, that is during the high-pressure period preceding the exams. A number of implications may be drawn from these findings considering that these specific mindfulness facets could be enhanced through specific programs for students to potentiate their capacity of managing anxiety and the many pressures associated with medical education. Although personality traits are relatively stable through time they are not cast in stone and can be enhanced, as research has shown, through specific interventions (for reviews and metanalyses see Galante et al., 2018; Gilmartin et al., 2017; Jayawardene et al., 2017; McConville et al., 2017; Quaglia et al., 2016; Spinelli et al., 2018).

This is in line with actions that are being currently taken in the context of healthcare education programs aimed at integrating student wellbeing issues into existing medical education curricula (Chung et al., 2018; Dobkin & Hutchinson, 2013; Kemp et al., 2019; Rau & Williams, 2016; Ripp et al., 2017). In this perspective, it is important to note that the concern for medical students’ wellbeing on the one hand challenges the notion of the medical profession’s traditional stoicism and culture of perfection whereby learners hesitate to disclose their need for help when in distress. On the other hand, as some critical voices have pointed out (Stergiopoulos et al., 2020), championing wellbeing as a core competency may inadvertently lead to enhancing the very culture of perfectionism it intends to dismantle. Present results add up to the world-wide consensus on the necessity to adopt concrete actions at the systems-level and by embracing universal design approaches that benefit all learners alike (Kemp et al., 2019; Ripp et al., 2017; Stergiopoulos et al., 2020). Medical school should be a place where students learn about themselves and their vulnerabilities and develop awareness of their inner experiences in parallel with gaining the necessary skills and knowledge to become a part of the future medical workforce (Colonnello et al., 2020; West et al., 2016). Cultivating awareness and nonjudgmental acceptance of one’s inner experiences is a crucial self-regulation resource that can help medical student enhance their wellbeing as they learn and build the necessary self-care skills that will sustain them throughout their high-pressure education and future professional careers.
